# An integer optimization algorithm for robust identification of non-linear gene regulatory networks

**DOI:** 10.1186/1752-0509-6-119

**Published:** 2012-09-02

**Authors:** Nishanth Chemmangattuvalappil, Keith Task, Ipsita Banerjee

**Affiliations:** 1 Department of Chemical Engineering, University of Pittsburgh, 1249 Benedum Hall, 3700 O’Hara Street, Pittsburgh, PA, 15261, USA

**Keywords:** Gene regulatory networks, Non-linear dynamics, S-system, Robust network identification, Bootstrapping, Integer programming, Optimization algorithm

## Abstract

**Background:**

Reverse engineering gene networks and identifying regulatory interactions are integral to understanding cellular decision making processes. Advancement in high throughput experimental techniques has initiated innovative data driven analysis of gene regulatory networks. However, inherent noise associated with biological systems requires numerous experimental replicates for reliable conclusions. Furthermore, evidence of robust algorithms directly exploiting basic biological traits are few. Such algorithms are expected to be efficient in their performance and robust in their prediction.

**Results:**

We have developed a network identification algorithm to accurately infer both the topology and strength of regulatory interactions from time series gene expression data in the presence of significant experimental noise and non-linear behavior. In this novel formulism, we have addressed data variability in biological systems by integrating network identification with the bootstrap resampling technique, hence predicting robust interactions from limited experimental replicates subjected to noise. Furthermore, we have incorporated non-linearity in gene dynamics using the S-system formulation. The basic network identification formulation exploits the trait of sparsity of biological interactions. Towards that, the identification algorithm is formulated as an integer-programming problem by introducing binary variables for each network component. The objective function is targeted to minimize the network connections subjected to the constraint of maximal agreement between the experimental and predicted gene dynamics. The developed algorithm is validated using both *in silico* and experimental data-sets. These studies show that the algorithm can accurately predict the topology and connection strength of the *in silico* networks, as quantified by high precision and recall, and small discrepancy between the actual and predicted kinetic parameters. Furthermore, in both the *in silico* and experimental case studies, the predicted gene expression profiles are in very close agreement with the dynamics of the input data.

**Conclusions:**

Our integer programming algorithm effectively utilizes bootstrapping to identify robust gene regulatory networks from noisy, non-linear time-series gene expression data. With significant noise and non-linearities being inherent to biological systems, the present formulism, with the incorporation of network sparsity, is extremely relevant to gene regulatory networks, and while the formulation has been validated against *in silico* and *E. Coli* data, it can be applied to any biological system.

## Background

The progress in the field of experimental techniques in systems biology in recent years has contributed significantly to the analysis and understanding of gene regulatory networks [1]. The simultaneous measurement of the expression levels of thousands of genes has become possible with these techniques. The time series data of gene expression obtained from the high-throughput techniques typically contain comprehensive information about the structure of the system. However, reverse engineering that data for identification of interactions between genes and reconstruction of the regulatory network is still a challenging problem.

A variety of modeling approaches have been developed recently for inferring genetic networks from gene expression data. Identification algorithms are dependent on how the network is modeled [2], and include Boolean logic [3,4], Bayesian [5-7], and information-theoretic approaches [8]. Several approaches use steady state information, the data of which typically coming from “structural perturbations” (such as gene knockout studies) [9], which might be difficult to obtain for some systems. Alternate approaches using time series data include dynamic Bayesian networks [10,11] and differential equation-based models [12,13]. Of the latter, initial reports on reverse engineering gene networks assumed linear model approximations [13,14]. While such approximations retain simplicity in the identification algorithm, it may be inadequate in predicting strongly non-linear systems. One way of representing non-linear gene dynamics is the S-system model, a power-law formulation which incorporates both production and degradation terms of the genes. Previous studies have looked into network identification of non-linear systems with the S-system [1,12,15-20], which presents a more challenging task than identification of linear systems. In addition to non-linearities, gene regulatory networks are highly noisy and stochastic [21] which can lead to difficulties during network inference. Therefore, a strong need exists for robust network identification of non-linear systems in the presence of high system variability, while also being able to incorporate relevant biological information.

**Figure 1:**
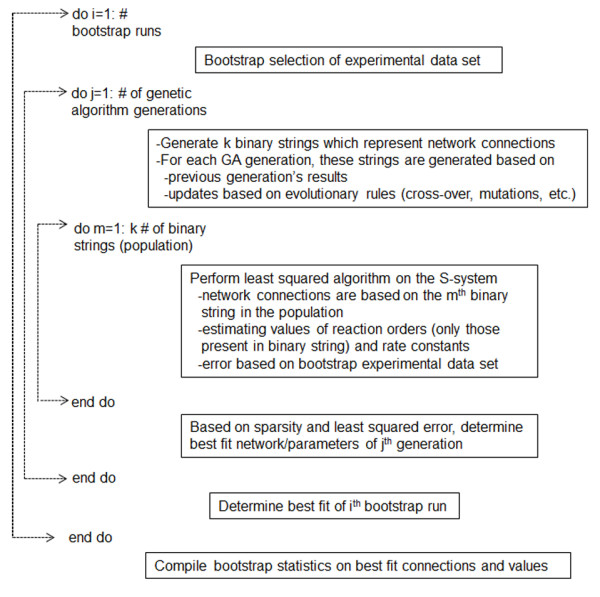
Pseudo-code of the robust network identification algorithm implementation.

In the current report, we model the dynamics of gene expression by S-system formulation. Upon doing so, we formulate the network identification algorithm as a bi-level optimization problem, governed by the hypothesis of network sparsity. Network sparsity has been experimentally observed in various biological systems such as the visual system of primates [22], auditory system of rats [23], and olfactory system of insects, to name a few. The sparsest gene network has also been eluded to be a robust one [24]. Governed by the hypothesis of sparsity of network connections, the target of our network identification algorithm is to find the network structure with minimum number of connections that is in agreement with the experimental data at an acceptable level of tolerance. We have earlier proposed an optimization formulation to identify the regulatory network from time profiles of gene expression data [25]. The previous algorithm was based on the following approximations: (i) gene expression dynamics were approximated by linear ordinary differential equations (ode); and (ii) the system was treated as deterministic by considering only the mean experimental data for the analysis. In the present algorithm, we developed a novel formulism which utilizes bootstrapping to identify robust networks from noisy data. The aforementioned approximations are removed by (i) representing the gene expression profile with an S-system model and (ii) directly accounting for variability in experimental data. Our algorithm, as detailed in the methods section and represented in Figure 1, enables identification of robust networks from an inherently non-linear and noisy system. We test the performance of our algorithm in various case studies including *in silico* and experimental data sets.

## Results

The performance of the developed bi-level integer programming algorithm is demonstrated on three case studies. In the first case study, we consider *in silico* gene expression data generated from a benchmark artificial 5-gene network model. In the second case study, the applicability of the algorithm on a larger network is tested using an *in silico* 10-gene network. In the third case study, the algorithm is applied to an experimental data set of the SOS DNA repair system in *E.coli.*

### I Case Study 1: Five gene network model

The purpose of this case study is to validate the algorithm on a small network with and without experimental noise. The chosen 5-gene network model [16] has been used as a benchmark problem by different research groups to test the validity of their algorithms [15,19].

#### *IA* Network identification without noise

**Figure 2:**
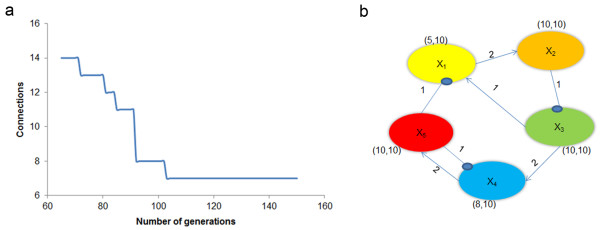
**Identification of a 5-gene network without noise.** (**a**) Convergence study of the genetic algorithm. The number of connections identified in each of the solutions generated by GA is plotted. No feasible solution was found with less than 65 generations. (**b**) Identified network. Arrows represent positive regulation and the filled circles represent negative regulation of the genes. Kinetic orders of each connection are represented above the corresponding connecting lines and the rate constants for each gene are shown above the genes. All connections and parameters are consistent with the original differential equations used to generate the *in silico* data.

Using the S-system formulation, the 5-gene network model can be represented by the system of five coupled nonlinear ode, shown in Additional file 1 as equation 1 [16]. In order to test our identification algorithm on this model we first generate *in silico* data by integrating these equations, which we use as experimental data for the identification algorithm. To formulate the bi-level optimization problem, *n*^*2*^ = 25 binary variables are introduced corresponding to each of the five connections. Genetic Algorithm (GA), used to solve the upper level integer programming problem, does not have a convergence criterion. Standard practice is to evolve the population for enough generations until no significant improvement is observed. Figure 2(a) illustrates the convergence characteristics of the GA for this example; at over 103 generations, the optimal output remained invariant. The efficiency of the algorithm depends on appropriate choice of starting population, as well as other involved parameters, in addition to the number of generations. The initial population size plays an important role in the quality and efficiency of the algorithm. A small population size may lead to local convergence or extremely large number of generations. To avoid that a population size of 20 was chosen and the algorithm evolved for 150 generations. The crossover probability is chosen to be at a standard value of 0.5, and the chosen mutation probability of 0.02 was expected to maintain diversity in population. Since the data contain no noise, the tolerance in the lower level least square optimization problem has been kept at a very low value (10^-5^). Typically least square optimization routines are very sensitive to the user defined initial guess. To make sure that the algorithm can identify the underlying network structure even without any *a priori* information, we deliberately assigned the initial guess values for the least square optimization problem to be largely different from the actual values, and tested the algorithm for various combinations of the initial guess.

Figure 2(b) illustrates the 5-gene network identified using the above formulation. The kinetic orders (*g*_*ij*_) are depicted over the connection and the kinetic rate constants (α_ij_, β_ij_) are depicted in brackets. The precision and recall value were both a perfect 1.0, indicating the accuracy with which the proposed algorithm predicted the network structure from time profile gene expression data. In addition, the identified kinetic orders and rate constants are also in agreement with the actual network model presented in Additional file 1 equation (1). These results validate the performance of the algorithm for a small network under deterministic conditions.

#### 1B Network identification under data uncertainty

**Figure 3:**
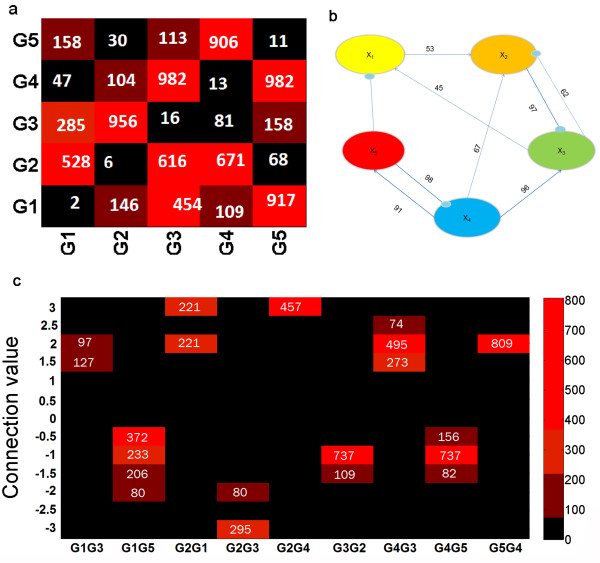
**Results from 5-gene network identified under data uncertainty with 5% noise. (a)** Number of bootstrap occurrences for each connection (1000 bootstrap samples total). **(b)** Identified network structure. Numbers above each connection represent percent occurrence, with the thick lines representing the number of connections appearing in more than 90% of the bootstrapped samples and the thin lines representing the connections appearing in more than 45% of bootstrapped samples. **(c)** frequency of specific connection values shown as heat map.

The performance of the algorithm is next analyzed in the presence of experimental noise, generated by adding 5% Gaussian noise to the time-course data generated from equation (1) shown in Additional file 1. Three different data sets are generated in this fashion to represent three experimental replicates of the samples. These three data sets are then resampled using bootstrapping to generate 1000 artificial data sets. The network identification algorithm was then applied at each of the data sets to generate 1000 alternate networks. The presence of noise in the data restricts the accuracy by which the predicted profile can agree with the data. Hence, the tolerance was relaxed to 0.12 and the GA code was evolved for 200 generations while retaining the population size of 20.The ensemble of alternate networks thus generated was analyzed for frequency of appearance of each of the connections (Figure 3(a)) which was hypothesized to directly correspond to its robustness against experimental noise.

Figure 3(b) further illustrates the identified robust network connections screened for 45% occurrence, with frequency of occurrence of network connections being depicted over the connection. Quite encouragingly, the algorithm correctly identified all the existing connections in the actual network. However because of noise, the algorithm also identifies two false interactions involving gene 2, hence resulting in a recall and precision of 1 and 0.78, respectively.

The expected values of the S-system parameters estimated at 90% confidence level are represented in Table 1 (*g*_ij_) and Table 2 (α_ij_, β_ij_), which demonstrates the excellent performance of the algorithm in identifying network parameters even from noisy data. While the error of the rate constants (compared to the actual values) is relatively high, it should be noted that the results of the network identification would not be as sensitive to these parameters as to the connectivity values, and therefore the rate constant values could vary significantly and not affect the gene profiles or the recall/precision. Furthermore, the error on the reaction orders (g_ij_) is very low, further demonstrating the accuracy of the network identification. The heat map in Figure 3(c) further shows the algorithm’s effectiveness in finding a tight range of reaction orders of the robust connections in the network.

**Table 1:** Comparison of the identified S-system reaction order values to actual values

**Connection**	**g**_**actual**_	**g**_**estimated**_
G1G3	1	1.2 ± 0.09
G1G5	-1	-1.0 ± 0.03
G2G1	2	2.4 ± 0.04
G2G3	NA	-3.4 ± 0.06
G2G4	NA	3.9 ± 0.05
G3G2	-1	-1.1 ± 0.03
G4G3	2	1.9 ± 0.02
G4G5	-1	-1.0 ± 0.01
G5G4	2	2.0 ± 0.02

**Table 2:** Comparison of the identified S-system rate constant values to actual values

**Gene**	**α**_**i**_		**β**_**i**_	
	**actual**	**estimated**	**actual**	**estimated**
X1	5	3.8 ± 0.2	10	18.0 ± 0.8
X2	10	13.8 ± 0.9	10	16.2 ± 0.2
X3	10	13.8 ± 0.2	10	11.2 ± 0.23
X4	8	8.1 ± 0.1	10	11.8 ± 0.1
X5	10	10.3 ± 0.05	10	8.9 ± 0.03

**Figure 4:**
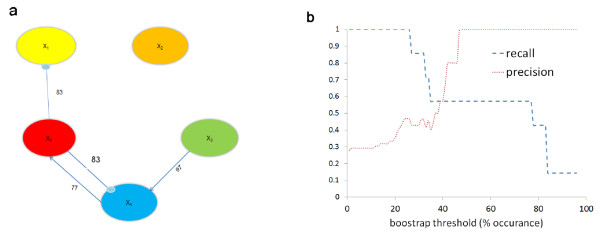
**Results from 5-gene network identified under data uncertainty with 10% noise. (a)** Identified network structure. Numbers above each connection represent percent occurrence. **(b)** Sensitivity of recall and precision to bootstrap occurrence threshold.

To evaluate the accuracy of the formulism under increased uncertainty, the algorithm was tested under various amounts of added noise. As one would expect, the accuracy of the algorithm depends on the level of noise added to the *in silico* data. Table 3 shows this trend, with the precision and recall being compared with 5, 7, and 10% noise. Increasing noise increases the number of false negatives, thereby reducing the recall. Interestingly, precision actually improves with increasing noise, indicating less false positives. This trend seems to converge, with both the recall and precision holding constant at 7 and 10%. Figure 4(a) shows the identified network with a data set incorporating 10% white Gaussian noise. The algorithm does not identify any connection which is not in the actual network (e.g. 0 false positives) and is therefore able to achieve a perfect precision. However because of noise, the algorithm also fails to identify three of the actual connections (false negatives), hence resulting in a recall of 0.57. It should be noted that the frequency threshold affects the results, and depending on the system, needs to be tuned. Figure 4(b) shows the sensitivity of the recall and precision to this threshold value.

**Table 3:** Effect of added noise on the network identification results

**Percent noise**	**Recall**	**Precision**
5	1	0.78
7	0.57	1
10	0.57	1

White Gaussian noise was added at different amounts to the five-gene network *in silico* data. Each of these data sets was used in the network identification algorithm, with their performance measured by the metrics of recall and precision.

**Figure 5:**
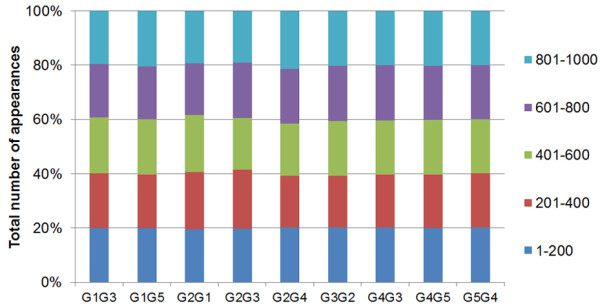
Convergence study on network identification results using bootstrapping with 5% noise.

While the analysis is performed on 1000 bootstrap samples, it is computationally expensive to solve 1000 network identification problems. Hence, we investigated the sensitivity of the identified robust network on the number of bootstrap samples by considering a broad range of samples from 200 to 1000. Figure 5 illustrates the percentage of total number of appearances of each identified interaction in every 200 bootstrapped samples, using 5% noise. The difference in the maximum and minimum number of appearances is less than 8% for all connections. The clearly shows that as little as 200 bootstrap samples can be enough in drawing statistically significant conclusions, which is in agreement with the literature [26].

#### 1C Deterministic network identification under data uncertainty

**Figure 6:**
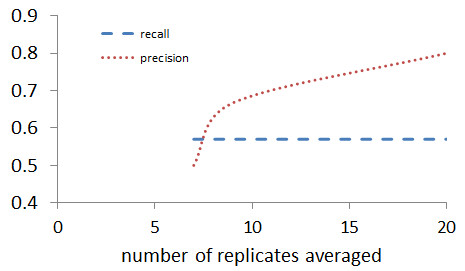
**Deterministic approach to network identification under noisy data.** Increasing number of replicates were generated using 10% noise from the *in silico* results, averaged, and used in the network identification algorithm, with their recall and precision quantified. Although three and five replicates were also used, these are not shown because no solution was found.

To assess the necessity of this bootstrapping technique, the aforementioned results were compared to a control group which did not utilize bootstrapping. To do this, a more deterministic approach was employed. Experimental replicates were generated as detailed: 10% white Gaussian noise was added to the 5-gene *in silico* network. Instead of bootstrapping these replicates, the deterministic network identification was performed on the mean of the replicates. This was done for 3, 5, 7, 9, and 20 replicates with the resulting precision and recall calculated for each case; results are shown in Figure 6. As shown, when the input data is generated from fewer than seven replicates, a solution is not found. Even with seven replicates, the results are relatively poor. While the recall is comparable to that generated from bootstrapping (~0.57), precision is much worse (0.5). As the number of replicates is increased, this precision increases; however, even at 20 replicates, precision is not perfect (0.8). Furthermore, in practice, generating this many experimental replicates is often not feasible. This illustrates that the proposed bootstrapping technique offers an accurate way of determining robust connections over a more traditional method, even with limited number of experimental repeats.

### II Case Study 2: Ten Gene Network Model

**Figure 7:**
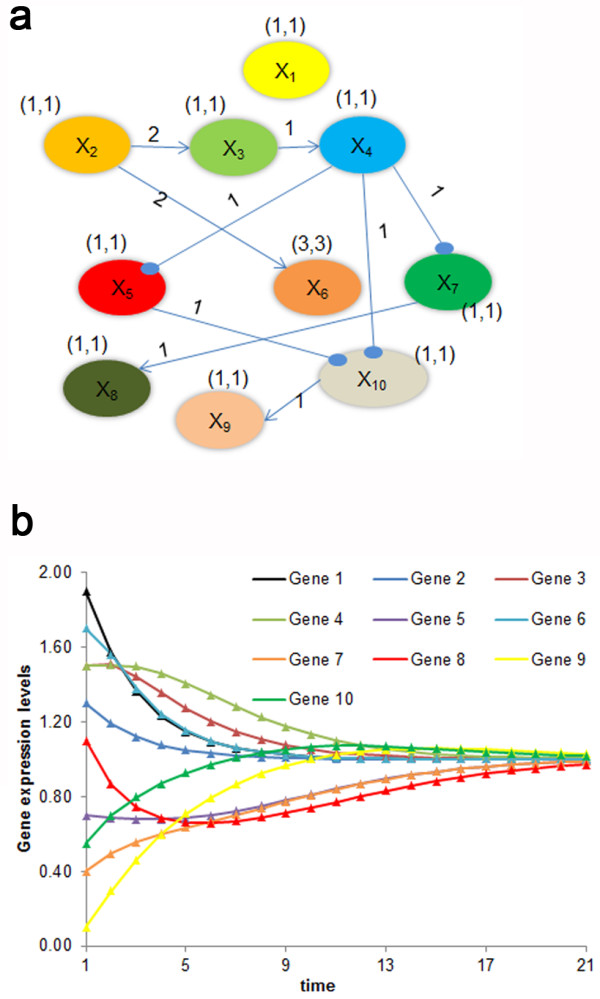
**Results from the 10-gene network.** (**a)** Identified network. Arrows represent positive regulation and the filled circles represent negative regulation of the genes. The kinetic orders of each connection are represented above the corresponding connecting lines and the rate constants for each gene are shown above the genes. (**b)** Time profile for the ten gene network. The triangles represent the profile generated from the *in silico* data and the lines represent the predicted profile.

In this example we investigate the performance of the developed algorithm in a larger network consisting of ten genes, as depicted in equation (2) shown in Additional file 1. For the deterministic case study the tolerance was specified at a low value of 10^-5^. Because the 10-gene network increases the number of binary variables in the upper level to 100, more GA generations are needed to obtain a converged solution; therefore, the number of generations was increased to 1000. The identified connections and kinetic parameters are shown in Figure 7(a), with the kinetic orders (*g*_ij_) depicted over the connections and kinetic rate constants (α_ij_, β_ij_) in brackets over the genes. The comparison of actual and identified time series profiles is shown in Figure 7(b). As evident from the figures, the algorithm correctly identified all the connections, kinetic orders and rate constants with a precision and recall of 1.0, thus verifying the satisfactory performance of the algorithm in larger systems.

### III Case Study 3: Experimental Data of E.Coli SOS DNA repair

The proposed algorithm is next applied to the SOS DNA repair system of *E.Coli*[27], based on the gene data measured by Ronen *et al.*[28] which is available online [29]. In this model system, the response to DNA damage is governed by a few key genes, which in turn regulate the expression of more than 30 genes which have specific roles in DNA repair. A proposed model is that the RecA protein binds to single stranded DNA, and this nucleoprotein is integral in LexA cleavage, a transcription factor which is a major regulator of the DNA repair genes [27]. The work of Ronen *et al.* investigates the Michaelis-Menten kinetic parameters associated with promoter activity for eight of the major genes in this system. Experimental kinetics were measured by first incorporating a GFP reporter plasmid for each of the gene’s promoter. DNA damage was induced, and the resulting GFP intensities were measured. The number of GFP molecules is proportional to the promoter activity, and can be taken to be analogous to the rate of transcription [28]. We therefore used this promoter activity data [29] to represent gene expression (with the experimental intensity data normalized by the mean column intensity) and used it in our algorithm. Among the four data sets provided by the authors, we chose the third and fourth for this case study because these are measured at the same conditions. Our objective was to identify regulatory interactions between six genes: *uvrD, lexA, umuD, recA, uvrA* and *polB*.

**Figure 8:**
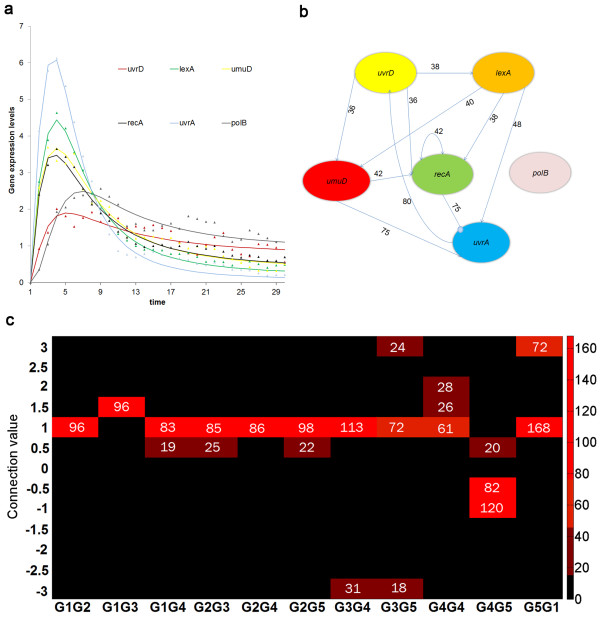
**Results from the 5-gene experimental*E.Coli*data.** (**a)** Time profile for the gene network, based off of the mean experimental data. The triangles represent the experimental data and the lines represent the predicted profile. (**b**) Identified network structure from experimental data for six gene system. The percentage of connections in the bootstrapping samples are marked on the connections. (**c**) frequency of specific connection values shown as heat map (connection coding: 1-uvrD, 2-lexA, 3-umuD, 4-recA, 5-uvrA, 6-polB).

Identification of this 6 gene network will require 36 binary variables; hence the GA parameters were retained similar to our first case study presented earlier: 20 populations evolved through 200 generations. The error tolerance, however, had to be relaxed to a higher value of .7 because of noise inherent in the experimental data set. Figure 8(a) compares the actual experimental data with the predicted profiles generated from the identified algorithm, which shows excellent agreement.

In the next step the robust connections of the identified network are further analyzed by bootstrapping the experimental data set. Since our previous analysis on the first case study demonstrated 200 bootstrap samples to be adequate, in this example we generated 300 artificial data sets from the original experimental repeats. The network identification algorithm was solved at each of the data sets to generate 300 alternate networks. The frequency of occurrence of each network connection is analyzed over the array of alternate network and connections appearing with over 45% frequency are considered to be robust. Figure 8(b) illustrates the predicted robust network for the *E. Coli* data set along with the frequency of repeat of each connection. The corresponding estimated kinetic orders (*g*_ij_) and rate constants (α_ij_, β_ij_) with 90% confidence level are shown in Table 4 and Table 5, respectively. The heat map in Figure 8(c) further shows how well the algorithm identifies a robust network .

**Table 4:** **Estimated reaction order values of the*E. Coli*SOS DNA repair network (connection coding: 1-uvrD, 2-lexA, 3-umuD, 4-recA, 5-uvrA, 6-polB)**

**Connection**	**g**_**estimated**_
G1G2	0.9 ± 0.04
G1G3	1.4 ± 0.03
G1G4	0.9 ± 0.04
G2G3	0.9 ± 0.04
G2G4	0.9 ± 0.07
G2G5	0.8 ± 0.08
G3G4	1.0 ± 0.03
G3G5	0.9 ± 0.03
G4G4	1.3 ± 0.07
G4G5	-0.7 ± 0.05
G5G1	1.0 ± 0.14

**Table 5:** **Estimated rate constant values of the*E. Coli*SOS DNA repair network (connection coding: 1-uvrD, 2-lexA, 3-umuD, 4-recA, 5-uvrA, 6-polB)**

**Gene**	**α**_**i**_	**β**_**i**_
X1	3.2 ± 0.28	8.4 ± 0.12
X2	1.5 ± 0.09	1.6 ± 0.19
X3	1.7 ± 0.21	1.5 ± 0.17
X4	5.3 ± 0.16	1.6 ± 0.07
X5	1.6 ± 0.16	2.0 ± 0.15
X6	4.3 ± 0.22	3.8 ± 0.12

## Discussion

In this work, we present an algorithm to identify robust regulatory networks from time profiles of gene expression data. Our identification algorithm is primarily developed on the hypothesis of sparsity of biological network connections. In our earlier work we established the validity of the hypothesis of sparsity using a simplified linear ode representation of gene expression dynamics in a deterministic system. Herein we further advance the algorithm by incorporating more realistic non-linear representation using an S-system formulation of gene expression dynamics. The identification algorithm is formulated as a bi-level optimization problem in which the upper level solves an integer programming problem while the lower level is a continuous parameter identification problem. Furthermore, we propose a framework to incorporate noisy experimental data towards identification of a robust regulatory network. This is done by first generating artificial experimental repeats using the bootstrapping technique, followed by solving the identification formulation at each of the bootstrap data sets. From this library of identified prospective networks we isolate the most-repeated network connections which we hypothesize to be a robust connection, having low variability to experimental noise.

The upper level integer programming problem is solved using GA. There are several advantages of using GA to solve the above problem, the most important being that it does not require gradient evaluation. This is a significant advantage for the above problem with non-linear ode as constraint function. In addition, GA starts its search not from a single point in the feasible parameter space, but from multiple locations specified in the starting population. Hence, it holds the chance of converging at global minima, although such convergence cannot be guaranteed with GA. However, it also suffers from the disadvantage of increased computational cost. All the computations reported here have been carried out on 2.66 Ghz processer and 16 GB RAM server. The computational time for the five gene network without noise was 1 hour and the same network with noise was 2.5 hours. The computational time for the experimental data was 3 hours. For the 10 gene network, the genetic algorithm needed more generations to converge, resulting in computational time of 11 hours. Hence, extension of the current solution procedure to a much larger data set will be expensive. While the same formulation will still be applicable in a larger system, alternate solution procedures are currently being investigated for its extension to larger networks.

In the formulation presented in equation (2), the only user defined parameter is the value of the tolerance which dictates how closely the model prediction must agree with experimental dynamics in order for the network to be considered in the overall algorithm. While for an *in silico* case study without noise the tolerance may not play a vital role, it will be relevant when evaluating noisy scenarios. Specifying a low tolerance value (10^-3^) in our algorithm under noisy data failed to identify any network, as would be expected. Moreover, using a low tolerance is not advisable when using data sets with noisy replicates since we are not targeting a profile which exactly fits the noisy data; the target is to identify network profiles which describe all the noisy scenarios relatively well. On the other hand, a relaxed tolerance runs the risk of compromised prediction quality. In order to quantitatively evaluate the effect of specified tolerance on the identified network structure, the bootstrap/ bi-level optimization algorithm was repeated on the same 5-gene dataset with different tolerance values. Table 6 illustrates how the precision and recall of the identified network changes with altered tolerance values. Quite interestingly it is observed that precision is relatively insensitive to the network tolerance, while recall worsens with increased tolerance. This is very encouraging since this implies that even with relaxed tolerance the identified network does not have false positive connections, although false negative connections increase. Increase in false negatives can be explained by the nature of the objective function which tries to minimize the number of connections. Hence, relaxed tolerance will always lead to a sparser network, as seen in Table 6. This analysis indicates that even for a relaxed constraint the algorithm may fail to identify all the connections but the identified connections will always be accurate with low probability of false positivity.

**Table 6:** Effect of error constraint on 5-gene network identification, 5% noise

**Error**	**Precision**	**Recall**	**Number of connections**
0.13	0.78	1.0	9
0.20	0.88	0.88	7
0.25	0.78	0.78	7
0.30	0.88	0.7	5
0.35	0.88	0.7	5

The performance of the developed robust identification formulation is illustrated using three different systems. The first two case studies are based on *in silico* data which allows for detailed analysis of the performance of the algorithm. Overall the algorithm was found to demonstrate excellent predictive capability both in the small 5-gene network along with larger 10-gene network. The proposed bootstrapping scheme was found to adequately capture the precise network from the noisy data as well. Encouraged by the *in silico* results, we applied our algorithm to dynamic experimental data of a 6-gene network responsible for DNA damage repair in *E. Coli*[28]. While verification of the identified network will be difficult for this system, the time profile of gene expression data predicted by the identified network is in good agreement with the experimental data set. A thorough literature search for existing knowledge of network interactions revealed that quite a few of the predicted connections have been reported in parallel studies. Our algorithm inferred the regulation of *recAumuD* and *uvrA* by *lexA*, which is consistent with the findings reported earlier [12]. Another interesting finding is that our results suggest that *polB* does not influence any of the other genes in the system (*pol B* does not up- or down-regulate any other gene), a finding which was also reported by Kumura *et al.* Furthermore, our identified network shows the self-regulation of *recA*. This protein is the main factor responsible for sensing DNA damage, and has been reported to promote the transcription of itself, thereby promoting damage recognition, and other repair genes [27,28].

The current approach offers an improvement on existing algorithms. Numerous studies have used the 5-gene network (the current case study I) to test the accuracy and efficiency of their network identification methods. A comparison between the methods is presented by Kimura *et a*l. [12] for the five gene network without noise. While most studies do not report the metrics of precision and recall, the accuracy of the results is still commented on. Most methods have a shorter computational time than the proposed method. However, our algorithm is able to predict a perfect network (recall and precision of 1), while the other algorithms deviate from this. Therefore, there is a trade-off between computational time and accuracy, and selection of the most appropriate method for the system of interest should be chosen judiciously. Nevertheless, this comparison shows that recall and precision are an improvement over many existing algorithms when analyzing the 5-gene network. Additional improvements could be made on the current approach to decrease computation time, such as parallel programming, or by altering the formulism (e.g. avoiding direct integration of the system of ode).

## Conclusions

These results show that our bi-level integer optimization algorithm is able to effectively identify the topology and connection strength of gene regulatory networks, even when the gene dynamics are non-linear and noisy in nature. By using the biological trait of sparsity, the algorithm optimizes the number of connections in the network while maintaining agreement in gene temporal profiles with the experimental input data. Even with uncertainty and noise in the data, something which is unavoidable on an experimental level, our bootstrapping/identification combination was able to identify a robust network. While we have demonstrated the effectiveness of our algorithm on *in silico* and *E. coli* data, its formulation, biological relevancy, and results are applicable to any gene regulatory network, as long as time-series data is available.

## Methods

### S-system representation of gene expression dynamics

Identification of the regulatory network from time series gene expression data first requires modeling the dynamic evolution of the individual genes constituting the network. Here we model gene dynamics as a set of coupled non-linear ode following the S-system formulation, which captures the non-linearity in gene expression profiles using a power-law kinetic representation.

For a system with N-genes, the S-system model can be represented using equation (1):

(1) $${\overset{\cdot }{X}}_{i}=\alpha_{i}\prod_{j=1}^{n}X_{j}^{g_{\mathrm{ij}}}-\beta_{i}\prod_{j=1}^{n}X_{j}^{h_{\mathrm{ij}}}$$

Where *X*_*i*_ is the concentration of the gene *i, α* and *β* represent the kinetic rate constants, *g* and *h* represent the kinetic orders for the production and degradation terms, respectively, and *n* is the total number of species in the system, in this case total number of genes in the network. In this work, we are using a modification of the above equation by assuming that species degradation follows a first order kinetics of the corresponding species and independent of other species (*h*_*ij*_ = 1 for *i = j*; 0 otherwise). While being relevant to biological systems [18], this assumption also reduces the unknown parameters from 2*n(n + 1)* to *n(n + 2)*[16]*.*

### Network Identification Algorithm

Our network identification algorithm is primarily based on the hypothesis of sparsity of network connections governing biological systems. Hence our overall objective is to determine the sparsest network which can satisfactorily capture the observed network dynamics. Following this idea, the network identification problem is formulated as an optimization problem with the objective of promoting sparsity given the constraint of maximizing predictive capacity. Such problem definition results in a bi-level optimization problem, where the constraint itself is an unconstrained optimization problem. In the current formulation using S-system to model the gene expression level (equation (1)), the kinetic orders (*g*_*ij*_) are decomposed into two parts: binary part, λ_ij_, which determines the existence of the connection; and continuous part, ρ_*ij*_, representing the nature and strength of interaction for an existing connection. A value of 1 of the binary variable λ_ij_ would indicate the presence of the corresponding connection $X_{i}\leftarrow X_{j}$, while value of 0 indicates its absence. These binary variables are optimized in the upper level which results in an integer programming problem. For each chosen network in the upper level, the connections are sent to the lower level, where corresponding ρ_*ij*_ are optimized to maximize network prediction and hence minimize deviation of the network predictions from the observables. The lower level essentially optimizes both strength (magnitude) and nature (sign) of the existing connections (ρ_ij_ , reactions orders) as well as the strengths of the production and degradation rate constants (*α*_*i*_*,* and *β*_*i*_ respectively). Hence it results in a continuous non-linear programming problem where the objective is to minimize the deviation of the predicted profiles from experimental data in a least square sense. A constraint of tolerance (*tol*) is imposed on this minimized error which defines the maximum allowable deviation in prediction. The mathematical formulation of the network identification problem in its entirety is shown in equation (2):

(2) $$\begin{array}{l} \emptyset =\min\sum_{i,j=1}^{n}\lambda_{\mathrm{ij}}\\ subjectto:\arg\min\chi (\lambda )\leq tol\\ where\\ \chi ={\left[\sum_{t=1}^{nstep}\sum_{i=1}^{n}{\left(x_{t,i}^{\exp}-x_{t,i}^{\mathrm{pred}}\right)}^{2}\right]}^{\frac{1}{2}}\\ \frac{dx_{i}}{dt}=\alpha_{i}\prod_{j=1}^{n}x_{i,j}^{g_{\mathrm{ij}}}-\beta_{i}\prod_{j=1}^{n}x_{i,j}^{h_{\mathrm{ij}}}\\ g_{\mathrm{ij}}=\lambda_{\mathrm{ij}}\cdot \rho_{\mathrm{ij}}\\ h_{\mathrm{ij}}=\left\{ \begin{array}{l} 1,i=j\\ 0,otherwise \end{array}\right.\\ 1\leq \sum_{i,j=1}^{n}\lambda_{\mathrm{ij}}<n\times (m-3) \end{array}$$

*λ*_*i j*_ = binary variable

$x_{\mathrm{ij}}^{\exp},x_{\mathrm{ij}}^{\mathrm{pred}}=$experimental and predicted gene expression levels, respectively

*α*_*i,,*_*β*_*i*_ = kinetic rates constants of ith gene's production and degradation, respectively

*g*_*ij*_*,h*_*ij*_ = kinetic orders of production and degradation, respectively

*nstep* = number of time points

*n =* number of genes constituting the network

*m* = number of experimental time points

In the above formulation *∑λ* represents the total number of network connections, minimizing which will promote sparsity in the network. The upper level integer programming is solved using combinatorial optimization techniques since combinatorial approach is known to handle *L*_*0*_ minimization problems more efficiently than approximation algorithms [30]. Of them, evolutionary algorithms are particularly efficient in finding a good approximate solution for combinatorial problems [31]. In this work, we have used genetic algorithm (GA) for solving the integer programming problem, while the lower level non-linear programming problem is solved using a standard least square optimization routine.

GA is typically designed to handle unconstrained optimization problems. One technique for constraint handling in GA is by penalty function, where the constraint is conditionally incorporated in the objective function. For conditions violating the constraint the objective function is penalized, and not so otherwise. In the current formulation the constraint is incorporated in the objective function using the following modification of the objective function:

(3) $$\varphi =\min\left[\sum_{i,j=1}^{n}\lambda_{\mathrm{ij}}+penalty*\frac{\max\left[\zeta ,0\right]}{\zeta }\right]$$

where $\zeta =\frac{\arg\min\chi \left(\lambda \right)}{tol}-1$

A significant advantage of the bi-level formulation is that it allows optimum utilization of experimental data by sequentially reducing the number of unknown parameters in the lower level. In a conventional least-square parameter estimation problem, the connectivity is fixed and includes all possible network connections. Therefore, the size of the identifiable system is restricted, governed by the availability of experimental data points so that number of unknown parameters is less than the number of data points. For instance, a single level algorithm, using the above S-System formulation, would be restricted to less than m-3 genes. However, in the current bi-level formulation, this restriction is relaxed. Because the number of network connections are first reduced in the upper level, the number of genes to be analyzed is not so restricted, with the only constraint coming from the connectivity:

(4) $$\sum_{i,j=1}^{n}\lambda_{\mathrm{ij}}<n\times \left(m-3\right)$$

Hence the constraint is imposed on the maximum number of binary variables assigned in the upper level, but does not constrain the total size of the analyzed network. Moreover, our primary objective being sparsity of network connections, the formulation essentially tries to minimize the number of connections assigned to 1. Hence, except for the very initial phase of GA evolution, the constraint defined in equation (2) typically does not become active, and never so in the final optimal solution.

### Identification of Robust Networks

Real world data typically contains noise due to experimental uncertainty and system stochasticity. Biological data are particularly notorious for its inherent heterogeneity and stochasticity [32]. Hence it is important to explicitly account for data variability in order to increase confidence in the predicted network. In the presence of large experimental repeats it may be possible to determine robustness of identified network by repeatedly solving the network identification problem at each of the experimental data sets and analyzing the connections which are heavily repeated. However, drawing statistically significant inference would necessitate a large data set which is impractical and infeasible.

An alternative to actual experimental repeats is to use bootstrapping. The purpose of this statistical technique is to estimate the distribution of the estimator around the unknown true value *θ*. However, instead of achieving this with a large number of individual replicates, bootstrapping utilizes resampling of the data. In this way, a large number artificial data sets can be generated from a limited number of experimental repeats. For each bootstrap run, data samples are randomly chosen, with replacement, from the empirical distribution, with the size of each artificial set being the same as the experimental set (e.g. if the experimental set has 20 data points, so would the bootstrap set). For each bootstrap, the estimators (e.g. mean, variance, or, as in the case of the current work, regression parameters) are calculated, and with sufficient number of resampled data sets, relevant statistical information, including confidence intervals, can be estimated [26,33].

In our algorithm, we are dealing with limited experimental data. Hence, following the above methodology, we generate a large artificial data set by repeated resampling of the limited experimental repeats. Once the bootstrapped samples are obtained, the network identification algorithm previously described is applied to all bootstrap data sets to identify a network corresponding to each. The network sets thus obtained is further analyzed to determine the frequency of occurrence of each connection in the entire set of identified networks. We hypothesize that frequent occurrence of network connections in the bootstrap samples indicate the insensitivity of the corresponding network to experimental noise, and hence claim that connection to be robust.

In order to quantify the quality of prediction of the proposed algorithm the measures of *recall* and *precision* are used, calculated as:

(5) $$\begin{array}{l} recall=\frac{TP}{TP+FN}\\ precision=\frac{TP}{TP+FP} \end{array}$$

Where: TP (True Positive) denotes the number of connections correctly captured; FN (False Negative) denotes existing connections which are not captured in the identified network; and FP (False Positive) denotes connections which are incorrectly captured in the identified network. Following the above equation: a low value of recall would indicate a more conservative estimate which is unable to capture many of the existing connections; a low value of precision will indicate prediction of incorrect connections not appearing in the actual network; and a value of 1 will indicate perfect network identification. The flow diagram of the overall network identification algorithm is shown in Figure 1.

## Competing interests

The authors declare that they have no competing interests.

## Authors’ contributions

NC, KT and IB developed the algorithm. NC and KT analyzed the data and performed output analysis. NC, KT, and IB drafted the manuscript. IB conceived of the study, and participated in its design and coordination. All authors read and approved the final manuscript.

## Supplementary Material

Additional file 1 **Additional Equations.** The two systems of ordinary differential equations shown in Additional file 1 were those used to create the *in silico* data for cases studies 1 and 2, respectively. Click here for file
