# Supplementary material for: An integer optimization algorithm for robust identification of non-linear gene regulatory networks
Source: BMC Syst Biol. 2012 Sep 2;6:119. doi: 10.1186/1752-0509-6-119 (PMC3444924; doi:10.1186/1752-0509-6-119)
Supplement: Additional file 1 — Additional Equations. The two systems of ordinary differential equations shown in Additional file 1 were those used to create the in silico data for cases studies 1 and 2, respectively. [file 1752-0509-6-119-S1.pdf]

Additional Equations

$$\begin{aligned}
\dot{X}_1 &= 5X_3 X_5^{-1} - 10X_1 \\
\dot{X}_2 &= 10X_1^2 - 10X_2 \\
\dot{X}_3 &= 10X_2^{-1} - 10X_3 \\
\dot{X}_4 &= 8X_3^2 X_5^{-1} - 10X_4 \\
\dot{X}_5 &= 10X_4^2 - 10X_5
\end{aligned} \tag{1}$$

$$\begin{aligned}
\dot{X}_1 &= 1 - X_1 \\
\dot{X}_2 &= 1 - X_2 \\
\dot{X}_3 &= X_2^2 - X_3 \\
\dot{X}_4 &= X_3 - X_4 \\
\dot{X}_5 &= X_4^{-1} - X_5 \\
\dot{X}_6 &= 3X_2^2 - 3X_6 \\
\dot{X}_7 &= X_4^{-1} - X_7 \\
\dot{X}_8 &= X_7 - X_8 \\
\dot{X}_9 &= X_{10} - X_9 \\
\dot{X}_{10} &= X_4^{-1} X_5^{-1} - X_{10}
\end{aligned} \tag{2}$$
